# Circling the sandwich: measuring food waste and its drivers in UK prepared sandwich manufacture

**DOI:** 10.1038/s41538-026-00948-8

**Published:** 2026-06-27

**Authors:** Alexander Moores, Dimitris Charalampopoulos, Adrian Williams, Kerry Whiteside, Paul Buxton, Ximena Schmidt Rivera

**Affiliations:** 1 Department of Chemical Engineering, Brunel University of London, Uxbridge, UK; 2 Department of Food and Nutritional Sciences, University of Reading, Whiteknights, Reading, UK; 3 Faculty of Engineering and Applied Sciences, Cranfield University, Bedford, UK; 4 Samworth Brothers Limited, Leicester, UK; 5 Centre for Food Policy, City St Georges, University of London, London, UK

**Keywords:** Environmental impact, Chemical engineering

## Abstract

Food waste reduction and reutilisation is paramount to meeting national Waste Directives and achieving the Sustainable Development agenda. Implementing Circular Economy principles provides opportunities for cost savings, new revenue streams, and sustainable resource management. Applying the Food Loss and Waste (FLW) Accounting and Reporting Standard, this study identifies and measures sources of FLW in the manufacture of prepared sandwiches, and explores reduction and reutilisation from a Circular Economy perspective. The examined process contributes to circularity, with a loss rate of 13%, of which 97% is valorised. Waste hotspots include product assembly and ingredient preparation, while operational reductions, design changes, and stakeholder collaboration for high-value side-stream management would further reduce FLW impact. Examining a complex food item at the product-level for the first time, this study provides an evidence base for FLW reduction through inventory and hotspot analysis, contextualising the current extent of circularity and suggesting areas for further development.

## Introduction

Food waste is a global issue, given that a third of the world’s food supply is wasted^1^. It is estimated that 733 million people faced hunger in 2023^2^, a figure that has been growing since 2015. Ending hunger is one of the core aims of the Sustainable Development Goals (SDGs)^3^, while food waste puts additional pressure on the world’s resources, given that its production is a major driver of global environmental change^4–8^. Eliminating current levels of food waste would prevent 8–10% of greenhouse gas (GHG) emissions^9^, and is recognised as a core enabler of providing adequate diets from a sustainable food system^10^. Given population growth trends and nutrition transitions, increasing the efficiency of the food system is a necessity as global food demand in 2050 is projected to rise to 60–110% above that at the start of the millennium^11–13^.

Set amongst wider trends of modernisation in the food system, attention has been turned to understanding the environmental impacts and food waste burdens of different food products and supply chains^14,15^. Given that increased choice and less cultural engagement with food preparation appears to be driving food waste in the retail and consumer spheres^1,16^, convenience or prepared foods are the focus of an increasing number of environmental studies^17,18^. Food waste is prevalent in both developing and developed countries, and is generally split into two types. Food loss, commonly occurring in production, processing, and distribution, is the removal of material from the food chain due to the separation of inedible parts or through unavoidable discarding of food unfit for human consumption, while food waste, more common in the retail and consumption stages, consists of the discarding of food that is suitable for consumption, conventionally framed as being due to behavioural or market factors^19^.

A recognition of the scale of the problem has led to an increase in publications on food waste in the last decade^20,21^ and in the uptake of voluntary accounting and declaration schemes by food companies^22^. However, overall data on food waste is limited, being often based on secondary data and lacking particularly in individual supply chains and products^23–25^. Despite the recognition of Circular Economy (CE) approaches in managing food loss and waste (FLW) on a policy level, few studies highlight the waste management practices and behaviours of food production from a CE perspective, given the priority on consumer and retail waste^26–28^.

This study fills this gap by reporting on the physical characteristics and management conditions of FLW through a structured examination of the quantity, types, and drivers of waste within the food manufacturing sector, together with economic and environmental implications. This work explores how commercial food waste is reduced and valorised through the CE approach by using the UK sandwich manufacturing industry, being an economic and cultural cornerstone of the UK food sector^29,30^, as an exploratory case study. This study identifies how much food waste is created in the manufacture of a typical sandwich product, and analyses the causation and utilisation of food waste, sharing best practices and offering recommendations for increasing circularity and sustainability.

FLW occurs in all stages of the food supply chain^31^. Conventionally, FLW is concentrated in upstream processing in developing countries, and in the consumer sphere in developed countries^1^, driven by differences in infrastructure and consumption patterns^16^. In the UK, total post-farm FLW rates are estimated at 9.5 Mt with 70% arising in households, 16% arising in manufacturing, and 12% arising in hospitality and food service^32^. The large variety of product offerings and high quality assurance standards are known to drive an increase in FLW in manufacturing and processing sectors^33^, while only 2% of waste occurs in the retail setting directly^32^.

The production value of global FLW is estimated at $1 trillion (2012 USD), increasing to $2.6 trillion when considering the environmental, social and economic impacts borne by society from its management and the unnecessary removal of embodied resources from the market and environment^34^. The majority of environmental impacts of FLW arise from the resources used in primary production^35,36^, while non-recovery waste disposal options (i.e. landfill) lead to significant GHG emissions, eutrophication, and toxicity impacts^37^. Production of FLW accounts for 8% of anthropogenic GHG emissions^38^; a quarter to a third of agricultural land^39^; and 23% of fertiliser use^40^. European FLW water footprint is comparable to its municipal consumption^36^, and has significant impacts on biodiversity loss^41,42^. Meanwhile, the impact of FLW is largely influenced by its composition^40^. Projected population growth and nutritional transitions through to 2050 may inflate the environmental impact of FLW to four times its 2010 level^43^. Eliminating hunger (SDG 2.1) and halving food waste (SDG 12.3) is integral to global development^3^. In addition, food waste reduction has potential benefits for SDGs 3, 14, 15 (Human Health, Life Below Water, Life on Land) due to emissions and biodiversity impacts of the nitrogen cycle^44^, as well as synergies surrounding better livelihoods and reduction in poverty^45^. FLW minimisation and reutilisation is core to the UK’s Food and Drink Pact (formerly Courtauld 2030 Commitment) to reduce food sector GHG emissions by 50% against a 2015 baseline^46^, and wider 2050 Net Zero commitments^47,48^. Movements to more broadly reorient production and consumption systems in order to reduce and recirculate wastes are enshrined in the UK’s Circular Economy Package and the EU’s ‘Closing the loop – An EU action plan for the Circular Economy’ legislation^49,50^, wherein FLW is a direct measurable indicator.

The Circular Economy (CE) movement brings together cross-sectoral shifts in production and consumption patterns that broadly aim to reorient the global economy away from the incumbent linear, extractive and pollutive ‘take-make-use-dispose’ model prevalent since the industrial revolution, into one where energy, labour and materials are recirculated within the system for as long as possible^51^, thereby satisfying material and societal needs while remaining within the carrying capacity of the natural environment^52^. CE approaches are founded on an elimination of the concept of waste^53^, and are enabled by new technologies and industrial arrangements on the micro, meso, and macro level^54^. CE contributes to environmental and economic dimensions of the SDG agenda^55,56^ and to climate change mitigation and adaptation through material and energy usage reduction^57^.

Within the food system, CE movements have focussed on the reduction of agricultural inputs through recirculating nutrients and increasing efficiency within the system^27,58,59^; increasing food intensity through better diets, consumption patterns and novel business models^60,61^; and product design focussed on eco-effectiveness^62,63^. FLW management practices are essential for organising and enabling the adoption of CE approaches in the food system^64,65^. Actions within FLW management align with the food waste hierarchy^66,67^ and material cascade loop model^68^. Prioritised practices include prevention through early intervention, redistribution for human consumption, and re-use for animal feed^69^. By-product transformation and supply chain cascading can retain the material and functional value of food items^70^, while nutrients can be recycled through anaerobic digestion or composting^71^. Energy recovery options include incineration, biofuel production, or, least favoured, landfill with biogas collection^37^. Studies consistently find that food waste reduction has larger environmental benefits than reutilisation^35,37,72,73^, suggesting the global food system is not yet at a point where increasing material efficiency comes with an increased environmental or social burden^34^. Governments enable CE efforts in the food system by supporting the development and legislation of waste management best practices^74^; subsidies and financial support^75,76^; and the facilitation of industrial interventions and collaboration^77,78^, leading to benefits for wider society through increased material efficiency and resilience of food systems^68^.

While increasing attention has been given to the measurement of FLW of specific food products and supply chains, few studies in the literature use first-hand direct measurement approaches^21,25^. Mass flow analysis (MFA) is a common assessment tool, enabling the combination of existing data sources to create food waste models. MFA has typically been used to examine industrial sectors^79–82^, while some studies have examined individual products. Amicarelli, Bux and Lagioia^83^ quantified waste in the Italian potato chip industry, Wyngaard and Kissinger^84^ examined bell pepper production, while Garcia-Garcia, Stone and Rahimifard^85^ examined a number of UK fruit and vegetable processors and a brewery, identifying main sources of waste and suggesting potential for improvements. Meanwhile, a number of studies have adopted primary data collection methods, including analyses of dairy processing operations and supply chains^86–88^; examinations of tomato processing operations^28,89^; and examinations of pasta and bread supply chains^27,90^.

Up until now, FLW studies have focussed on food staples or minimally processed food products. With the increasing reliance of contemporary diets on convenience food products such as ready-made sandwiches and ‘meal-deal’ offerings^91^ and the large market share of this sub-sector^92^, it is important to understand the amount of FLW created in the manufacturing stage, wherein component ingredients are assembled into saleable food products, and to understand how such operations can utilise the CE approach within the context of wider circular food supply chains. While some previous studies have examined the environmental burdens of meals and complex food products^18,93,94^, detailed considerations of FLW generation and amounts were not included. Thus, the authors of this work are not aware of any prior literature targeted at quantifying the food waste burden of complex food items on a product level, meaning the characteristics of FLW generation in their manufacture and the implications for CE implementation have hitherto been unknown.

As such, this study seeks to fill this knowledge gap through addressing three main research questions: (i) how much FLW is generated in the manufacture of commercially-prepared, chilled ready-to-eat sandwiches, (ii) what are the major causes and resulting fates of lost material, and (iii) how are CE principles used in the FLW management in commercially prepared sandwich manufacture to reduce FLW volumes and impacts.

Being the first study to examine the food waste burden of contemporary sandwich manufacture, this work’s field increases the available data for food products of increasing importance to contemporary food provisioning practices. Furthermore, facilitating the exploration of FLW within supply chains, regions, and organisations helps food system actors better understand the volumes, location, and drivers of waste generation^22^. FLW accounting processes are integral to FLW policy design, enabling stakeholders to mark baselines and set targets for reduction^95^.

The remainder of this paper is structured as follows: section ‘Results’ presents an inventory of food waste arisings in the manufacture of the specific sandwich product selected for analysis—the roast chicken salad sandwich; section ‘Discussion’ discusses and contextualises the obtained results, and offers concluding remarks and future research directions; while section ‘Methods’ describes the methods and materials used in the study.

## Results

This section presents the inventory of types, volumes and fates of FLW as generated in the examined production system: the manufacture of chilled, ready-to-eat chicken salad sandwiches, as made by a prominent UK producer. The FLW inventory is presented, covering production for the period of January–September 2023, followed by an analysis of the causes of FLW, associated destinations, sustainability impacts, and the implications of potential future scenarios.

**Fig. 1:**
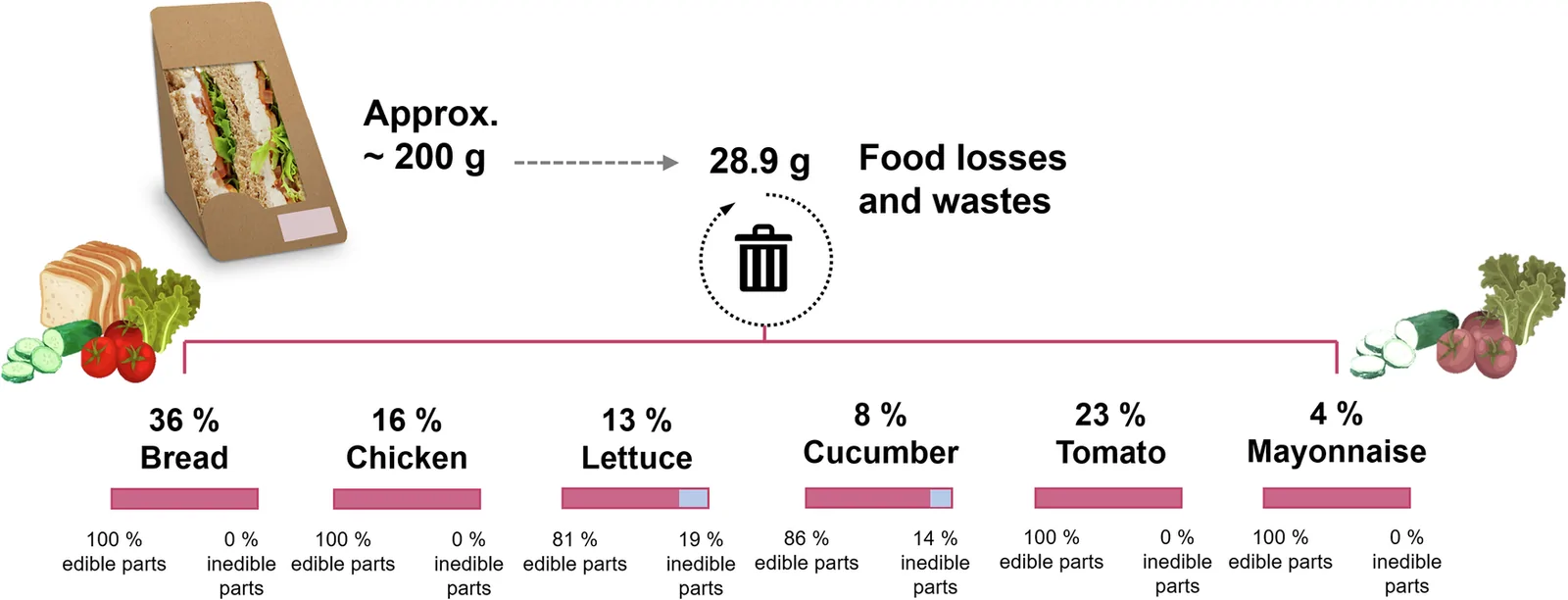
Volume of food loss and waste (FLW) generated during the production of a ready-made chicken salad sandwich, as normalised to a single unit. Image credit: Caziopeia^159^.

Across the 9 months of chicken salad production runs covered in this study, over 100,000 kg of ingredients were brought into despatch roughly 90,000 kg of finished sandwiches, or 470,000 units. A total of 13,500 kg of FLW was generated in the relevant manufacturing activities: made up of approximately 13,000 kg of edible food components and 502 kg of inedible parts, this corresponds to a loss rate of 12.7% of total material handled for the product. An overview of the ingredient splits of this material is displayed in Fig. 1, and the manufacturing stage splits are displayed in Table 1.

**Table 1:** Inventory results for the 9 months of production of chicken salad sandwiches, describing food and inedible parts removed from the sandwich manufacturing process stages

Production stage	Total FLW (in kg)	Total FLW distinguished in:	
		Edible parts	Inedible parts
Ingredient intake	68.0	67.2	0.9
Storage	166	165	1.2
Preparation	2010	1510	500
Assembly	10,400	10,400	0.0
Despatch	905	905	0.0
**Total**	**13,503**	**13,001**	**502**

As most ingredients arrive at the facility in a pre-processed state, 96.3% of the total FLW consists of edible parts. Associated inedible parts, including parts of salad items, make up 4% of total FLW. Of the total FLW volume, 35.5% was discarded bread slices, 22.6% was discarded tomato slices and whole tomatoes, 15.8% was discarded chicken slices, 13.3% was discarded lettuce, 8.4% was discarded cucumber, and 4.5% was discarded mayonnaise. Approximately 12,600 kg, or 93.3%, of the FLW was discarded as individual ingredients prior to or during sandwich assembly, while 905 kg, or 6.7%, was discarded in the form of finished sandwiches. Taking the average sandwich to weigh approximately 200 g, there is 28.9 g of FLW produced per sold sandwiches; roughly 14.5% of the product’s mass as sold. Figure 1 displays these results normalised to a single sandwich.

**Fig. 2:**
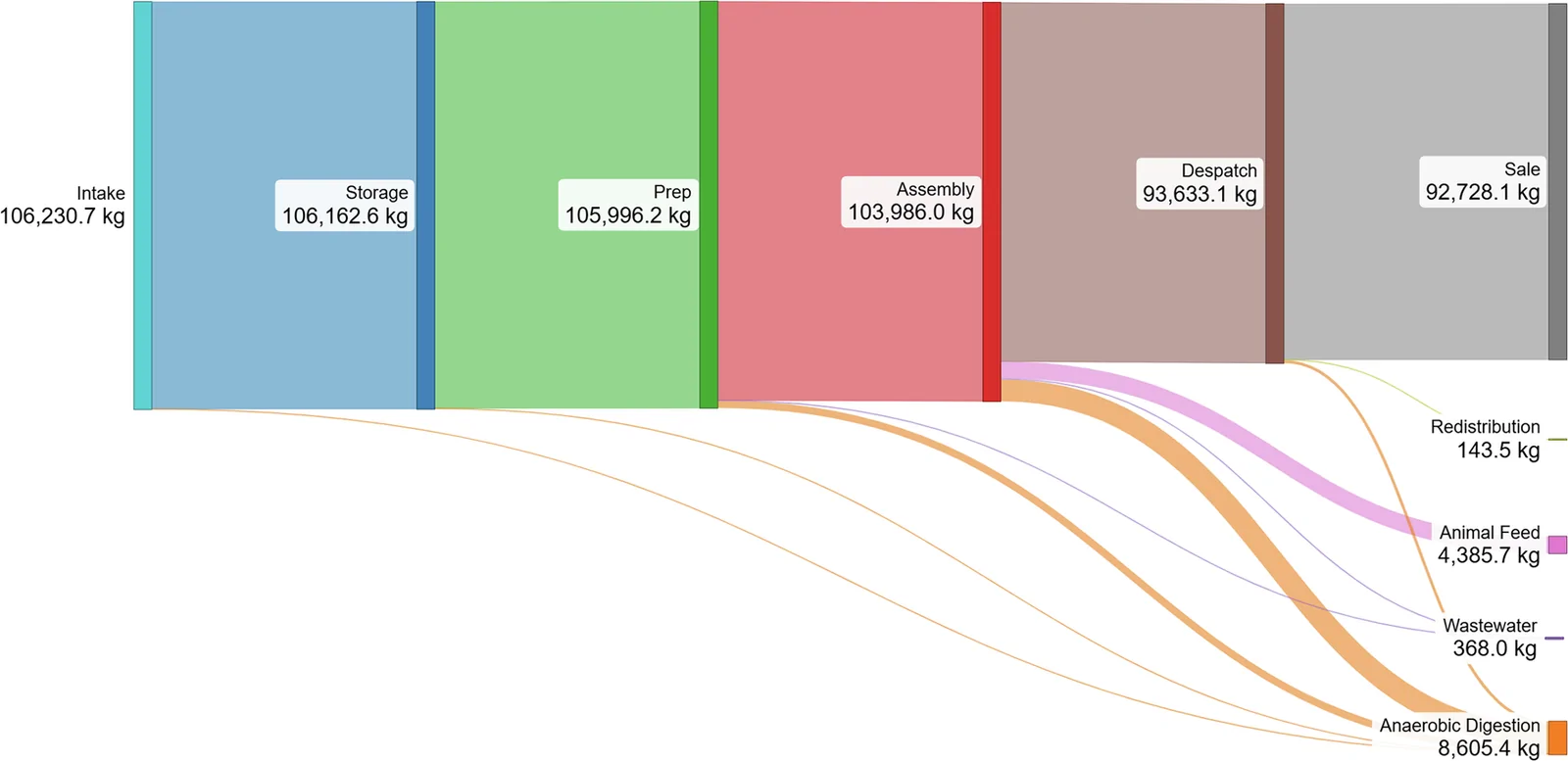
Mass flows of food material and FLW through the manufacturing operation. Includes all ingredients and inedible parts bought in for chicken salad sandwiches over the examined production period.; waste flows include edible and inedible parts.

FLW arising in the manufacturing operation is sent to four waste management destinations, in quantities shown in Table 2. A mixed solid waste stream totalling 63.7% of the FLW, comprising of mixed ingredient waste contaminated by or including animal proteins, is sent to a regional waste management company that co-digests the FLW with agricultural by-products, producing biogas and a nutrient-rich digestate fertiliser. A segregated waste stream totalling 32.5%, consisting of discarded bread slices and loaf ends, is sent to animal feed processors for re-use within the human food chain. The liquid and semi-liquid fraction, 2.7%, leaves the site in wastewater processing, primarily consisting of mayonnaise and the liquid parts of salad items. The remainder, 1.1% of the FLW, consisting of assembled sandwiches deemed unfit for sale (falling short of retailer standards or manufactured in surplus to order volumes) and ingredients for discontinued products, are redistributed for human consumption through donation to charities or sale in the manufacturing facility’s staff restaurant. A summary of overall mass flows is displayed in Fig. 2.

**Table 2:** Destinations of food loss and waste after leaving the manufacturing facility

Destination	Weight of FLW, kg	% of total
Redistribution	144	1.1%
Animal feed	4390	32.5%
Anaerobic digestion	8610	63.7%
Wastewater	368	2.7%
**Total**	**13,503**	**100%**

Material flows for each manufacturing stage, and destination of each waste stream are shown in Fig. 2, while a summary of the main causes of FLW arisings are shown in Table 3.

**Table 3:** Summary of the largest sources of FLW arisings in the sandwich manufacturing operation and causes thereof, totalling to 95% of FLW

Type of FLW arising	% of total FLW	Stage arising	Cause of arising
Bread loaf ends and rejected slices	32.5%	Assembly	Quality standards/expectations
Assembly processing losses	31.9%	Assembly	Ingredient processing
Salad trimming^a^	11.7%	Preparation	Ingredient processing
Rejected salad ingredients	9.9%	Assembly, Preparation	Quality standards/expectations
Rejected finished sandwiches	3.6%	Despatch	Quality standards/expectations
Sandwiches and ingredients taken for microbial testing	3.4%	Despatch, Preparation, Storage	Hygiene
Overproduction of sandwiches and prepped ingredients	1.7%	Despatch, Preparation	Planning and management
Supplier contamination and rejects	0.2%	Intake	Hygiene, upstream factors

^a^Note: Salad trimming, comprising of lettuce, tomato and cucumber parts, is made up of 41% inedible parts, while all other streams are solely edible food parts.

Over the five main production stages, the majority of FLW, 76.7% of total, arose during the product assembly stage, wherein prepared ingredients are booked out onto daily production runs, and sandwiches are assembled through a variety of manual and machine-aided steps. Within this stage, the majority of FLW is attributed to two processes. Firstly, before sandwich assembly, the ends of bread loaves are separated from the rest of the useable loaf; combined with slices rejected due to deformation or excess holes, this makes up 32.5% of total FLW. Secondly, a similar amount of FLW, 31.9% of the total, arose from ingredient mishandling or equipment-related spillage. Falling to the floor or directly or via the conveyor belt end, these processing losses cannot be reused to safeguard against potential contamination. With all ingredient types represented, 74% of processing losses occur during ingredient loading, 5% during sandwich cutting, and 21% during the packaging of sandwiches into their card-based sleeves. In addition to these factors, a significant volume of individual ingredients, or 8.2% of total FLW, is rejected during assembly after a visual assessment by line operatives. For example, cucumber and tomato slices whose diameters are smaller than a certain threshold are discarded at this stage due to visual tolerances set by customer requirements.

Within the ingredient preparation stage, a further 14.9% of FLW arises during steps where ingredients are removed from packaging and processed into a form ready for assembly. The source of FLW in this stage was salad trimmings: 11.7% of total FLW, consisting primarily of inedible parts removed from lettuce, tomato and cucumber, but including some additional edible parts due to over-trimming. This included a large contribution from lettuce cores and outer leaves (1050 kg); salad trimmings, while uncontaminated at the point of arising, are managed through the facility’s aggregated mixed FLW stream.

A small amount of FLW, 1.7% of the total, arose in the ingredient intake and storage stages. The majority of this waste consists of ingredients or products taken for hygiene sampling purposes, with a small amount of food also being rejected due to quality or contamination concerns. This material is commonly disposed of at the site and is not returned to the supplier.

The remaining 6.7% of FLW occurs in the despatch centre, where individual sandwiches are grouped and packed into larger volumes for shipping to retailers. FLW at this stage arises due to quality control checks and the removal of samples for testing, with a small amount of overproduction. Production runs are generally operated on a small surplus to avoid order shortfall, while excess units are taken to the staff restaurant to be made available to purchase by employees or donated to food redistribution charities.

**Fig. 3:**
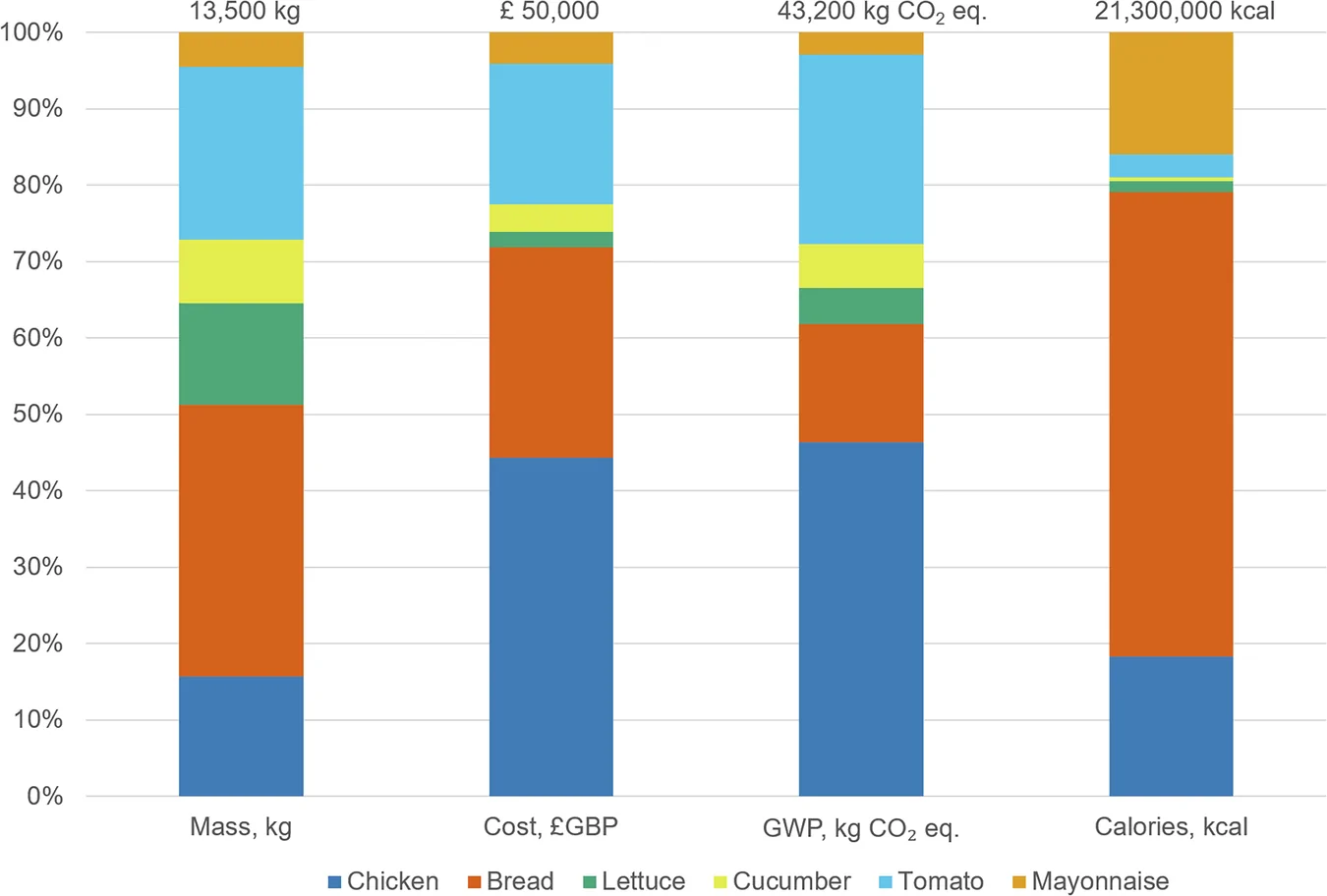
Contribution of each ingredient to FLW expressed in weight, cost, embodied greenhouse gas emissions, and calories removed from the manufacturing operation. Note: greenhouse gas emissions are expressed in terms of Global Warming Potential (GWP) in kilograms of CO_2_ equivalents.

The results of ingredient contribution analysis and impact assessment are shown in Fig. 3, relating material losses to sustainability indicators of cost, embodied GHG emissions, and calorie content. Taking into account the economic value of ingredients in the waste streams, the operation incurred a 9.7% loss rate, while the embodied GHG emissions contained in ingredients discarded in the waste stream made up 10.4% of the GHG emissions in the total material input. The percentage of total calories brought into the operation that were lost to the waste stream was 9.0%.

On a mass basis, the ingredient that had the largest contribution to the food waste burden was bread, followed by tomatoes, chicken, lettuce, cucumber and mayonnaise. However, when considering the economic value and embodied GHG emissions contained in components discarded in the FLW stream, contributions from higher-cost chicken and tomatoes become more important, in the ranges of 44.5–46.6 and 18.4–24.9% respectively, leading to a total loss of £50,000 in ingredient costs and a burden of 43,200 kg CO_2_eq. solely from unused edible parts. When considering calories wasted, bread dominates (60.8%) with marginal contributions from chicken (18.3%) and mayonnaise (15.9%), leading to a total loss of 21,300,000 kcal to the waste streams.

While the majority of organisational and policy food waste reduction targets focus on weight, the direct contribution to climate change, and the removal of calories and value from the market through FLW is important, especially in considering the aims of CE being to keep value and nutrients in the system for as long as possible. In the UK, chicken sandwiches have a market share of 36.6%^18^, while roughly 3.8 billion sandwiches are purchased each year^96^. It is estimated that the entire food manufacturing industry produced 1,500,000 tonnes of food waste and surplus in 2022^32^. If it is assumed that the manufacturing practices of the manufacturer under study are representative of the wider industry, this suggests that around 40,000 tonnes of FLW arise each year due to the manufacture of chicken salad sandwiches alone, or 0.03% of total sector FLW.

**Fig. 4:**
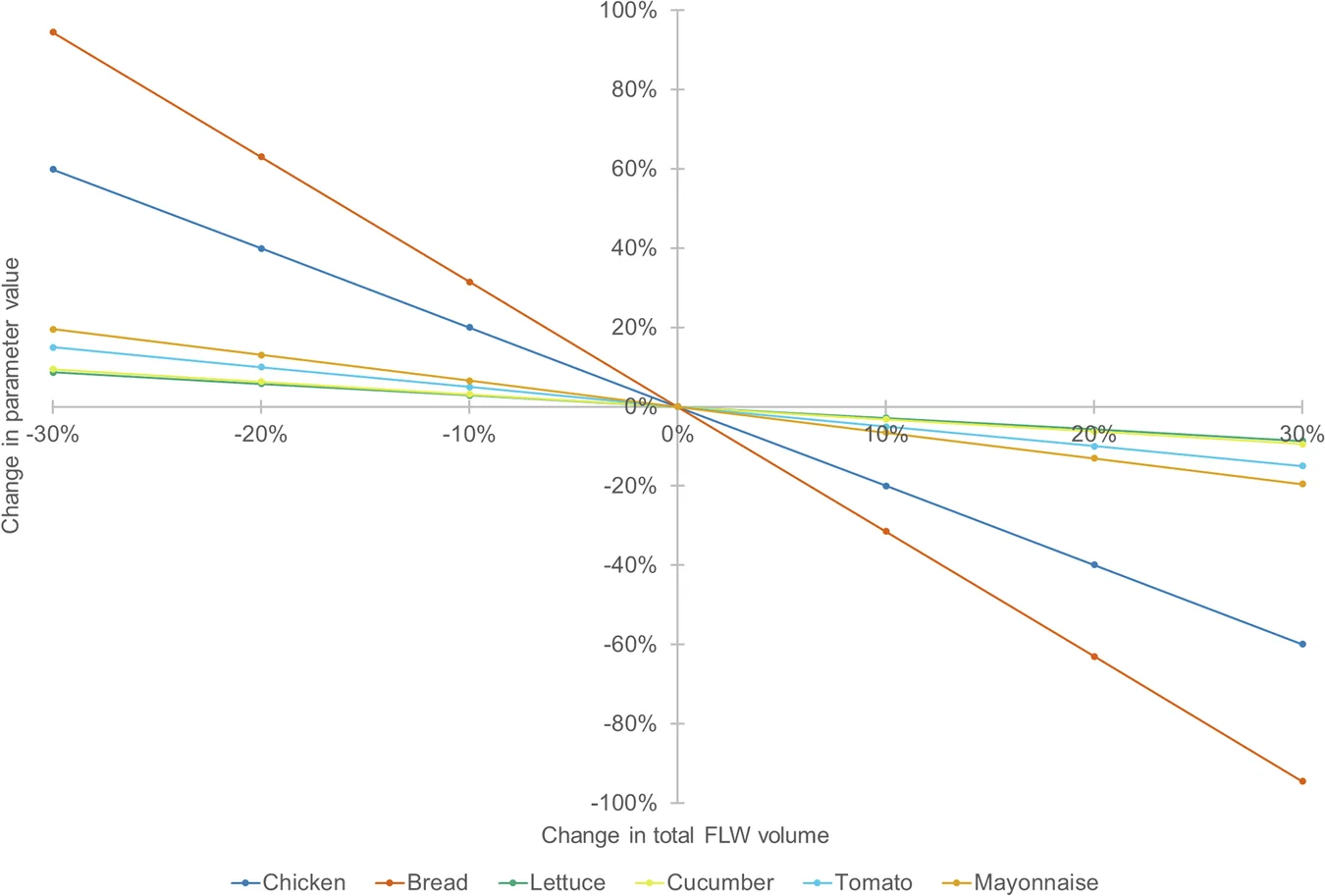
Parametric sensitivity analysis. The sensitivity analysis illustrates how changes in recipe usage amount for each ingredient leads to changes intotal FLW volume output by the inventory model.

To explore the robustness of the FLW inventory’s underlying model with respect to parameter uncertainty, a sensitivity analysis was undertaken modulating the expected ingredient utilisation in the assembly stages. Figure 4 shows how variations in the usage parameters affect the total FLW recorded for the product system. A moderate increase (+10%) in chicken and bread usage results in a large decrease in FLW volumes (between −20% and −30%), indicating significant sensitive explained by their larger contribution to final sandwich weight. Other ingredients, including salad components and mayonnaise, show a weaker influence on the total FLW volumes, given their relatively smaller contribution to the finished product.

As part of the scenario analysis, five feasible situations were explored modelling the results of interventions relating to the major causes of FLW identified in the inventory, evidence from prior literature, and current working avenues in the facility. A full description of scenarios is present in Table 6, while Table 4 shows the numerical results of this analysis.

**Table 4:** Exploring scenarios to reduce waste and raw material requirements

Scenario	Raw material reduction (%)	Waste produced (kg)	Waste reduction	Waste rate	Raw material saving (£)	Raw material bill change (%)
A	-	13,500	-	12.7%	£ -	-
B	−2.0%	11,400	−15.9%	10.9%	£10,600	−2.1%
C	-	9290	−31.2%	9.1%	£ -	
D	−2.1%	11,300	−16.7%	10.8%	£7800	−1.5%
E	−4.1%	4930	−63.5%	4.8%	£18,400	−3.6%

All scenarios are modelled on an output of 92,700 kg sold sandwiches (Scenario C and E also produce an additional co-product of 4200 kg and 4180 kg of bread ends).

The three cases of individual interventions highlight that significant FLW reductions can be made through improvements to internal operational processes and external dynamics. The largest individual FLW saving results from the valorisation of bread ends (Scenario C), leading to 4200 kg of waste being avoided. In this case, the same amount of material would be available for use in an alternative valorisation scheme. Both Scenarios B and D lead to an FLW reduction of 15.9–16.7% (2150 and 2080 kg, respectively), and in these cases, this leads to a raw material reduction of the same amount. Individually, these efforts reduce the FLW rate from 12.7% to 9.1–10.9%. If all efforts to be implemented concurrently, as illustrated in Scenario E, the waste rate would decrease to 4.8%, representing a 63.5% reduction in total FLW volume. In the combined Scenario, the 4.1% reduction in raw material requirement would save £18,400 in raw material costs, representing a 3.6% cost reduction, with additional savings likely to be made in reduced waste management costs and the potential for monetisation of the co-product stream.

## Discussion

FLW analyses contribute to cleaner production through the identification of hotspots for loss in food supply chains, thus providing evidence for policy design and direct intervention from interested stakeholders. Up to now, there is a lack of reporting of FLW generation in food manufacture and processing in the academic literature^33^, particularly from a CE lens^27^.

The analysis of the examined operation showed that the manufacture of chicken salad sandwiches contributes to CE in the UK food supply chain through the diversion of the vast majority of waste stream material, 97.3%, to re-use and nutrient recovery schemes, and through continued waste reduction efforts. The product system outperforms the UK average FLW treatment, which sees 19% sent to landfill and only a small amount to preferred management routes of human and animal distribution, 8%^32^. At the same time, the waste rate of the product system, 12.7%, appears significantly higher than the UK manufacturing sector at large. In addition, with CE frameworks emphasising the recirculation of materials at the highest possible value, opportunities for further improvement are present.

Using data from 2010 and 2021, Espinoza-Orias and Azapagic, IGD and WRAP estimate UK manufacturing FLW rates between 3.3 and 3.8% of total material handled^18,97^, while voluntary industry reporting initiatives in the ready-to-eat sector declare rates between 8.3% and 8.9%^98,99^ for the year 2019. Similarly, the business examined in this work’s overall organisation FLW rate was 5.1%^100^, suggesting there may be significant heterogeneity between individual products, product categories, and businesses within the sector. Product-level heterogeneity is also seen in the retail sector^101^, and has been attributed to varying shelf-life of ingredients, product complexity and differing assembly processes^101,102^. The significant contribution from assembly processes and trimmings seen in this work is in line with literature expectations, given the inclusion of fresh ingredients and intricate product formulation.

Comparing the results with other stages within the ingredient supply chains, a 2019 analysis suggests that FLW rates in the wheat bread cultivation, milling and baking processes are 60%^90^. However, FLW is dominated by inedible farm losses left on the field, used in animal feed or energy recovery. Taking account solely of edible material, the processes lose 6.4% of total edible parts, while downstream retail and consumer stages induce a further loss rate of 7.8%. Thus, the loss rate of bread in this manufacturing scheme, 10.7%, adds a significant burden to the loss of bread in the upstream supply chain. Fresh fruit and vegetables are generally acknowledged as high-waste foods, with tomato loss rates of 42% estimated globally^103^, rising to 56% in some studies in developed countries^89^. 2015 data suggests the rates of lettuce loss are similar in magnitude, up to 38% of harvestable mass, driven by both physical deterioration and unfavourable market conditions^104^. Losses in the retail and consumer sphere can be as high as 28% for fresh fruit and vegetables^105^ (2008 data), suggesting that the vegetable loss rates seen in this production scheme, approximately 31%, add a significant hotspot to an already high-loss supply chain.

With regard to the 28.9 g of FLW arising per sandwich, it was shown that the majority was caused by quality standards and expectations, both of prepared components and finished products, and by losses during assembly processes. Underlying drivers behind these include consumer expectations of the product, hygiene factors, technical limitations, and handling of ingredients. The remainder of FLW was attributed to product safety requirements, production planning, and a small amount of operator accidents and upstream contamination. Taking Barthel and Maxnaughton’s definitions^19^, 5.8% of the FLW can be considered unavoidable losses, consisting of inedible parts or legally mandated hygiene samples; 32.1% of the FLW can be considered possibly avoidable food losses, being ingredient parts that were originally edible, but made unfit for human consumption during processing stages; while the remaining 62.1% can be considered theoretically avoidable wastes, due to being edible parts discarded while still fit for consumption. Thus, there is a large potential for further improvements with regard to FLW prevention and reduction.

Jagtap and Rahimifard found a similar range of causes for FLW in their study of a UK ready-meal manufacturer. Following a 6 month intervention period, the most significant causes of FLW were over-producing, at 28% of FLW, followed by allergen-related spoilage (18%), expiry of components (16%), trimmings/offcuts (14%) and quality failures (13%)^101^. The overall manufacturing scheme in the ready-meal site resembled closely that of this work, made up of good intake & storage, preparation, assembly, packing and despatch processes. Yet, comparatively, this work saw a significantly higher proportion of FLW occurring due to assembly spillages and handling, and from the bread offcuts stream. The ready-meal factory had a significantly lower overall loss rate (1.92%), suggesting that the complexity in the assembly process of chicken salad sandwiches, i.e. hand-laying ingredient portions onto a flat bread surface as opposed to machine-filling trays with rice and sauce, is a significant driver for FLW as compared to the ready-meal examined in the prior work. On top of this, the bread waste stream adds a significant FLW burden. To a certain extent, the high FLW rate in assembly is constrained to a greater degree by product design and ingredient choices necessary for producing a sandwich matching in form and appearance to a homemade version, than from handling behaviours or processes on production lines. Alternative product formulations and ingredient choices may be able to reduce FLW through selecting less spillage-prone fillings^102^. Thus, this suggests that the product design stage may be a key leverage point for assembly-related FLW reduction.

In a more direct sense, perceived consumer expectations also lead to FLW arisings as a result of the rejection of prepared ingredients and finished sandwiches failing visual quality control, namely, out-of-size cucumber and tomato slices, and finished sandwiches with a poorly segregated cut-face. While more extensive changes to product design, including the use of bread ends in typical products, may require significant collaborative and process-related efforts, relaxing of product visual specifications could, by itself, lead to a significant waste reduction. There is an increasing appetite for lower-standard products in contemporary markets such as ‘wonky veg’ sales^106^, informed by increased consumer conscientisation on FLW^107,108^. It is generally accepted that the consumer sphere incurs the greatest source of loss in food supply chains^1,19^. Yet, examining the causes of FLW in this manufacturing scheme indicates that even some contribution within the manufacturing stage is caused by consumer or retailer expectations and behaviours. Thus, it is important for cross-supply chain stakeholders to collaborate in whole-supply chain reductions, due to the interaction of different stages in FLW causation^109,110^. Production trials, the earliest stage at which FLW performance may be understood, generally take place late in the new food product development process, once recipe development and ingredient sourcing have taken place^111^. This analysis suggests that greater collaboration between retailers, consumers, and manufacturers surrounding product design and specification could yield significant benefits throughout the food product’s lifespan.

From a Circular Economy perspective, the food manufacturing sector is seen as having favourable intrinsic recoverability of FLW material, due to existing intensive management practices and suitability of the food material for immediate consumption^112^. However, manufacturers face several barriers to recovering material and implementing valorisation processes.

Currently, the majority of FLW arising in the present operation is diverted to anaerobic digestion (63.7% of total), doing so at a higher rate than the UK food sector at large, according to 2018 estimates^32^, which sees a majority sent to landfill and incineration. Other companies in the UK convenience and prepared food market report similar proportions sent to anaerobic digestion^98,99^, with alternatives of human and animal redistribution also in use. Both general CE principles, favouring reduction, re-use, recycling and recovery, and food material specific frameworks report nutrient and energy recovery strategies such as anaerobic digestion as a beneficial alternative to landfill, yet are at the lowest rung in the hierarchy^67,113^. In this product system, expanding the redistribution of FLW is constrained by legislation preventing the re-use of animal protein-containing materials in the human food chain^74,114^. Thus, increasing the value of recirculation routes would require the separation of meat-containing material, or the identification of other material recycling and industrial use schemes. An additional FLW handling process can incur labour and material costs, presenting a barrier for implementation^115^, especially when recovered material may be low in volume^116^. However, they are essential for contributing to wider CE goals of recirculating material at the highest value^68^.

Teigiserova et al. provide further guidance on the selection of waste material for industrial use and bioconversion^67^. Inedible parts are most preferred, being otherwise not-preventable, followed by edible parts subject to uncontrolled degradation. The final priority material is high-burden food items, which themselves should be the first priority for FLW prevention. Conversely, consideration of the environmental and economic benefits of waste-derived products is important for the adoption of CE and waste management practices^53^. Recirculating FLW at the highest possible value is important in this manufacturing scheme, given the high cost of ingredients and calories contained in the waste stream. Due to its readily accessible sugar content, bread is increasingly recognised as a versatile feedstock for bioconversion^117^. Some industrial initiatives are available for reincorporation of bread waste into food products, such as in the substitution for malt barley in beer brewing^118^ and reformulating into flour for bakery products^119^. Additional conversion routes are being explored to produce ethanol^120^, protein products and concentrates^121,122^, and a variety of high-value biochemical products^117^. Mixed food wastes, while more challenging to valorise due to moisture content and inconsistent composition^123^, also present opportunities for bioconversion, including bio-ethanol production^124^, and animal feed via insect larvae production^125^, which has the additional benefit of circumnavigating legislative barriers for animal protein utilisation. At the same time, the low-technology readiness level of alternative routes presents a barrier for short-term adoption. Joint ventures, an alternative CE business model, can circumnavigate logistical barriers in the valorisation of fast-spoiling material such as bread and meat, by reducing transportation requirements^116,117^. Previous authors suggest that while requiring increased organisation and coordination between stakeholders, joint ventures can also reduce the burden of economic investment and pool technical resources^116,126^. This is found in FLW redistribution initiatives as well as bioconversion and industrial use^115^, suggesting it could be a valuable proposition for a range of possible CE strategy improvements.

Thus, while a significant gap in the production system’s use of high-value material recycling CE strategies is present, the business shows engagement with CE principles through current practices. Implementing waste reduction and utilisation actions facilitates the company in narrowing resource flows through material efficiency improvement, and closing resource flows through cascading waste material into biological cycles^127^. Landfill is avoided, in keeping with the overarching aim of CE to eliminate the concept of waste^53,54^. Meanwhile, the company’s existing FLW reduction strategies have already seen decreases in overall company FLW by 17% to 2019^100^, in part due to its participation with sectoral FLW and impact reduction movements Champions 12.3 and The UK Food and Drink Pact^22,46^. At the same time, aligning with wider CE goals requires additional actions. The Ellen Macarthur Foundation’s CE formulation includes the elimination of waste, recirculation of materials at the highest possible value, and the regeneration of natural systems. The food waste hierarchy and other frameworks provide guidance for the selection of higher value recirculation routes, while additional focus on product and process design may help address wider goals. Further prevention of FLW is found as the most significant environmental burden reduction strategy in existing evaluations of CE strategies through minimising the need for primary production and upstream processing activites^121^. Yet, additional guidance for food manufacturers promotes the integration of regenerative, diverse and upcycled ingredients into ingredient selection and product design^63^. Understanding how such provisioning practices impact overall costs is a significant challenge^128^. However, evidence that consumers value satiety in lunch-time products and are increasingly concerned with healthy choices suggests the possibility of synergies, providing a potential link to the additional CE conceptualisations of slowing resource flows and maximisation of consumer functional value^127,129^, aspects comparatively less explored in food contexts and biological cycles^116,130^.

Significant outcomes from the impact assessment and scenario analysis suggest that there is a considerable opportunity for cost savings, embodied GHG and calorie loss reductions from FLW prevention. The most significant raw material savings came from the scenario which explored changes to processing losses, followed by that exploring adjustments to visual standards. This provides further evidence for the importance of integrating FLW considerations in product and process design alongside retailer and consumer priorities. Meanwhile, the varying characteristics of the incoming ingredients led to different sustainability hotspots when social, environmental, and economic indicators were considered. The analysis indicated that targeting the FLW reduction of specific high-impact ingredients, such as animal proteins, may have a larger environmental benefit than reducing overall FLW weight. Thus, there is evidence that further CE implementation aligns with core economic sustainability priorities for the business, illustrated by the potential for raw material savings and new revenues, offering incentives for managerial decision-making towards these aims. Given that product design and visual specifications are a main cause of a large proportion of FLW, further improvements rest on collaboration with downstream supply chain stages. Coordination and maintaining partnerships with other stakeholders can additionally help food manufacturers find and utilise valorisation routes for surplus food and FLW streams^115^

While existing policies and governance provide recommendations for FLW reduction, re-use and redistribution practices^46,67,74^, industry surveys suggest a minority of businesses consider their waste management completely effective^131^. In addition to implementation costs and operational disruption, legal responsibility over the consumption of donated food presents barriers to effective action^16,132^. New technology provides alternative pathways, such as feed conversion via insect larvae production^125^, yet legislative change is identified as an intervention to facilitate wider management practices. An additional important contribution of this work is further evidence for the interconnection of FLW causation across supply chain stages^25,33^. 2011 and 2018 analyses indicate shares of EU and UK FLW occurring in the processing & manufacturing sub-sectors are between 16 and 24%^80,133^, yet this work suggests the actual responsibility of the manufacturing sector may be lower. Richter and Bokelmann argue that government can play a part in stakeholder collaboration through facilitating and legislating for communication and data sharing^134^, while Thyberg and Tonjes^135^ propose financial incentives for redistribution alongside data sharing platforms between supply chain stakeholders, with a particular emphasis on demand forecasting, as effective policy levers. While industry data sharing initiatives with respect to carbon footprinting and Life Cycle Assessment are increasingly well established^136^, food waste data exchange is as yet less prevalent^21,25^. Further, this analysis also put forward evidence for heterogeneity in the burden of different FLW materials, based on the varying impacts of ingredient supply chains. While some national and sector-level commitments integrate volume and GHG targets^137^, many simply target overall mass reductions^22,137^. Reaching consensus on the specific priorities for sustainable development can be a barrier to industrial action and evaluation^138^, suggesting a role for governance in setting clear targets for supply chains.

Within a research context, industry, academic and policy partnerships for knowledge sharing and technology development present opportunities for enabling wider adoption of underexplored CE pathways^62,113^. Full-scale impact assessments of waste management practices, for example, using the Life Cycle Assessment methodology, are important in the identification of whole-supply chain impacts, trade-offs and burden shifting when considering alternatives^121,139^. Within this work, the impact assessment considered GHG emissions, ingredient calorie content and raw material price as an indicator set, while additional indicators can evaluate the impact of FLW on other sustainability concerns. For example, utilisation of resources, impact on human health, and ecosystem effects are included in many Life Cycle Impact Assessment methodologies^140–142^. Of additional relevance to food products could be the use of protein content or nutritional scores as a social indicator representing wider health and nutrition aspects^143,144^.

While proximate causes of FLW generation were recorded and explored during inventory building, illustrating the major causes of FLW arisings, the apparent complexity and high number of underlying factors indicate that further analysis could explore the root causes of FLW in further detail. Systems mapping and broader social research methods have been given rising attention in the exploration of complex phenomena for problem solving and systems change, particularly in the food system, where technical and social factors interact^130,145^, and could help elucidate further pathways of causation, potential interventions, and barriers and enablers for their adoption.

The validity of the inventory model rests significantly on the assumption that stock movements and FLW arisings are recorded accurately by staff. The model also has some sensitivity to ingredient usage parameters, particularly with respect to those for bread and chicken, given these ingredients make up the majority of the finished product’s weight. While there is some evidence of underreporting of FLW volumes in retail settings^146,147^, in this model, the mass-balance approach minimises the risk of unrecorded wastes missing from the inventory. There may be some risk of the potential incorrect allocation to yield and processing drivers, as any unrecorded stock write-offs in other areas would be captured by these labels. Further interviews with operational and managerial staff could help to understand if this issue is prevalent in the examined product system. Sample assembly run data, which underpins the recipe usage values, indicated a low variation across the two sampled batches, less than 2%. While additional sample batches may help to remove further uncertainty, quality assurance procedures within the sandwich assembly process already take place regularly, both for raw ingredient preparation activities and in the amount used for each sandwich. Line operators use weighed depositor equipment with sample checks every 15 min for heavier items, while salad items are generally deposited by slice or handfuls. Given these factors, the overall dataset is likely to be accurate across the production period.

Being the first study to explore and quantify the food waste burden of complex food manufacturing at the product-level, there is a limited amount of data on similar product systems to compare against. Further, some sector and regional data may be at risk of being outdated given the infrequency of high-level surveys^21^. Increasing the number of food waste inventories in the literature would help both to contextualise the contribution of the individual product and the sandwich supply chain to FLW within the food manufacturing sector and to provide individual benefits to the decision-makers within those product systems. Further, given the context of increased modernisation and distancing of impacts within the food system, broadening the availability of product-level analyses would also provide valuable evidence for policymakers and consumers in the selection and encouragement of sustainable food provisioning practices^135^.

The generation of surplus food puts additional pressure on natural resources, the environment and human health; while the unnecessary removal of food from the human food system contributes to food insecurity, malnutrition, and hunger. This paper answers calls for the further exploration of food waste in food processing and manufacturing using quantitative and qualitative methods^33^. Being the first such study to inventory the food waste of a complex food on the product-level, it provides evidence for the significant food waste burden of the manufacture of prepared sandwiches as part of wider efforts to more greatly understand waste within the food system^1^. Yet, the manufacturing operation contributes to CE, participating in waste valorisation efforts through the adoption of waste hierarchy principles. At the same time, opportunities for improvement have been identified. The major drivers of food waste within this operation were found to be related to product visual and sensory standards and product design-related processing losses. As such, avenues for further progress include work into consumer expectations and product technical design, higher value by-product valorisation and material efficiency improvements. Significant opportunities for further waste reduction and utilisation have been identified in line with wider CE goals, illustrating the need for collaboration between retailers, manufacturers, consumers, and other partners across the supply chain, with policy support for its implementation.

While constrained by the limitations described, this study provides a justification for the wider research into food waste generation and management practices within complex food manufacturing contexts. In particular, broader analyses of types and drivers of FLW across the food system would be beneficial for the identification of further impact reduction efforts. For industrial stakeholders, this case study provides an example of the application of food waste quantification methods in a practical setting and highlights potential waste reduction and reutilisation practices arising from stakeholder collaboration. For policymakers, this work suggests that furthering understanding of the contribution from individual sub-sectors and providing support for data and material exchange across food supply chains could be effective waste reduction mechanisms, in alignment with wider food system transformation and Sustainable Development commitments.

## Methods

As well as facilitating policy design and interventions, the measurement and reporting of best practices within FLW management helps stakeholders to reduce FLW in situ^112^. Given the rising attention to the environmental impacts of convenience food^18,93^, this study design centres around the case study of UK commercially-prepared sandwich manufacture as a rich empirical context to explore FLW practices and management in a highly optimised food manufacturing environment.

In order to fulfil the research questions, the Food Loss and Waste Accounting and Reporting Standard (hereafter referred to as the FLW Standard) is adopted to structure a quantitative enquiry and assessment of FLW^148^. The FLW Standard is ‘a global standard that provides requirements and guidance for quantifying and reporting on the weight of food and/or associated inedible parts removed from the food supply chain’^148^. The FLW Standard guides cross-sectoral entities in the selection of individual measurement and reporting techniques to formulate representative and systematic FLW inventories. The FLW Standard consists of a formal scope setting stage, wherein the reporting entity declares the material type quantified in the inventory, being food material or associated inedible parts, or both; in addition, the timeframe of the study, the waste management destinations relevant to the product system, and the modelling boundaries. These definitions were used in the scope setting part of this study; a summary is reported in Table 5, according to which the FLW inventory relating to the manufacture of a typical ready-made sandwich product was built. Following scope setting, relevant data collection methods are chosen, executed, and the results analysed to create the FLW inventory, as described in the remainder of this section.

**Table 5:** Scope of FLW inventory

Timeframe	Material types and unit of analysis	Destinations included	Boundaries
January 2023 to June 2024	Total weight of food and inedible parts required to produce a finished sandwich (200 g)	Animal feed Co/anaerobic digestion Sewer Other: Redistribution within the human food chain	Food category: Sandwiches/Filled Rolls/Wraps (Perishable), (GPC Brick 10000255 CPC 23997, GFSA 16.0) Lifecycle stages: Food manufacturing stage (Manufacture of other food products n.e.c., ISIC 1079) Geography: UK (UN Code 826), single manufacturing site in Leicestershire, with UK-wide distribution chain Organisation: Single organisation Samworth Brothers Ltd.

The aim of this specific study is to quantify, describe, and analyse the FLW generated within the studied product system of roast chicken salad sandwich in an individually packaged, refrigerated and ready-to-eat format, as produced in a typical manufacturing scheme in the UK. The food waste inventory was prepared from primary data relating to the manufacturing operation, combining the sandwich’s main ingredients, which consist of sliced bread, frozen chicken breast slices, lettuce, sliced cucumber and tomato, and mayonnaise. This product was selected as chicken is the most common filling in commercial sandwiches in the UK^18,92^. In addition, the chicken salad sandwich contains a range of paste-like and solid-discrete ingredients, which have been shown to contribute differently to losses^149^. Further, its manufacture uses a range of processing techniques. Therefore, the exploration of this product provides insight into waste generation problems indicative of and experienced by the sandwich manufacturing industry as a whole.

The production scheme is based on a leading sandwich manufacturer based in the UK. The manufacturer produces high-quality sandwiches and similar products for the food-to-go and entertaining market, supplying leading UK retailers and employing around 700 people. The core sandwich assembly process involved five main actions: ingredient placement, topping, cutting, stacking, and packing; while additional ingredient handling and management stages include ingredient intake, storage, and preparation, and the arrangement and despatch of retailer orders. Within the facility, the business also undertakes planning and management, commercial, and development activities.

The FLW Standard outlines 10 preferred ways of collecting data and quantifying waste amounts to produce an inventory^148^. This work used a combination of approaches, including direct measurement, approximation, and inference by calculation, to create an inventory meeting the study scope and fulfilling data quality requirements of transparency, accuracy and relevance within the practicalities of data collection in a large and complex manufacturing environment. Core data for the inventory consisted of primary data collected from the manufacturer in January 2023 to June 2024, while secondary data, including GHG emissions, were used for further analysis stages.

Stock movement records were used to calculate the FLW produced in intake, storage, ingredient preparation and despatch stages, capturing production activities taking place between January and September 2023. Operatives use manual scales to record ingredient write-offs and weight changes through preparation processes. Where ingredients are shared across multiple products made by the manufacturer, FLW arisings were allocated to the examined product on a mass basis, given that upstream activities are identical for all sandwiches produced in the facility.

Mass balance was used to calculate the FLW generated in the product assembly stage. Stock records capture allocated ingredient batches and unused components, while a significant amount of waste occurs during assembly through line processing losses. Ingredient processing losses were calculated by comparing the net amount of ingredient booked into production lines and an expected ingredient utilisation in the finished products, as per Eq. 1.

1 $${w}_{i}=({p}_{i,{in}}-{p}_{i,{out}})-{{Ne}}_{i}$$

Where, for ingredient *i*,

*w* = processing losses,

*p*_*in*_ = amount of ingredient prepared,

*p*_*out*_ = amount of prepared ingredient not used,

*N* = number of sandwiches manufactured in the examined time period,

*e* = expected ingredient utilisation in finished product.

While manufacturer specifications state an acceptable range of ingredients used per sandwich, a sample of production batches, including the assembly of 4520 sandwiches, was examined to verify the ingredient usage amounts, *e*, through a waste composition analysis and mass balance conducted in June 2024. Sampled through two typical production runs of standard order volume and duration, the mass balance was conducted by recording the number of units produced, the net mass of ingredient booked to the production line, and manually separating and weighing the ingredient fractions in the waste stream. An assumption was made that mayonnaise adhered primarily to the bread slices in order to facilitate estimation of its recipe usage. Further details on assembly stage sample data and recipe usage values can be found in the Supplementary Information, Table S1.

The prepared FLW inventory combines the stock movement and mass balance data sources and assigns waste arisings to the four relevant waste routes outgoing from the facility in alignment with observed facility practices: redistribution to humans, diversion to animal feed, anaerobic digestion, and wastewater treatment. FLW in the wastewater stream includes residual mayonnaise from equipment cleaning, with residual solid wastes being recovered through filtration and diverted to other streams. The inventory includes both food and associated inedible parts, with edibility ratios taken from the National Nutrient Database for Standard Reference^150^, while other non-food waste materials, such as packaging, are excluded. Weight changes due to the addition or removal of water are also not included, given the minimal processing and rapid turnaround: as such, the inventory reflects the condition of the FLW at the manufacturing facility gate.

Supplementing the base inventory, an exploration of the causes of FLW was conducted, examining the reasons for FLW arisings across the manufacturing stages. Site visits took place before, during and after the core data collection phase, to conduct line walks and observations using the Gemba method from lean manufacturing systems^151,152^ and to facilitate the reviewing of stock records that capture stock reasons for write-offs. Waste in each stage was grouped based on the types of arising and the underlying causes, allowing for quantification across ingredients and stages. Process maps and identified causes were verified through consultations with operational and managerial staff to ensure accuracy and completeness of the analysis.

To explore wider sustainability impacts of the FLW generated and explore the potential for cost savings and environmental and social burden reductions, an impact analysis stage was conducted, quantifying the loss of components in terms of environmental, economic, and social indicators. Given the food system’s significant contribution and sensitivity to climate change^8,153^, embodied GHG emissions were selected as an environmental indicator. The GHG emissions of raw ingredient production uses the ClimateHub database^154^, utilising relevant entries for frozen chicken, prepared bread and mayonnaise, greenhouse cultivated tomato, lettuce and cucumber and open field black pepper production, representing typical UK market mixes. The ingredient GHG inventories include primary production, processing, packaging and transport, with impact characterised using the IPCC GWP100 impact assessment methodology. Given that the purpose of the analysis is to explore the contribution of different ingredients and potential impact on savings, end-of-life treatment is not included. For the economic indicator, ingredient purchase price was selected, given its dominance in manufacturers’ bottom lines and the downstream effects on retail prices^155^, while ingredient calorie content was selected as the social indicator, due to the importance of satiety to consumers of food-to-go lunch products^129^ and the impact of calorie loss on food security^16^. Ingredient cost and calorie content data were collected from leading wholesale UK distributor catalogues, and the UK Department for Rural Affairs, Environment and Agriculture’s weekly wholesale fruit and vegetable dataset for the year 2024^156,157^. In order to maximise decision-making value, production costs and embodied GHG emissions were allocated on an economic basis between inedible and edible parts, in line with prior work^158^. Inedible parts hold an allocation factor of zero, given that these parts are unavoidable from an FLW reduction perspective. As an additional analysis stage, FLW impacts were scaled across the sector’s overall production, assuming similar impacts across manufacturers, to contextualise against national and sector-wide FLW impacts.

Finally, sensitivity and scenario assessments were conducted to identify the robustness of the results towards variations in process data that carry uncertainty, as well as to explore changes to the FLW inventory from potential future interventions. While the inventory model uses direct measurements for the majority of waste sources and is thus considered high data quality^148^, the assembly processing stage uses a mass balance derived from estimated ingredient usage arising from a sample of production runs. As such, to test the impact of variation in this sample data, inventory results were recalculated based on parameter alterations within the range ±30%, selected to express reasonable degrees of variation in ingredient loading while retaining practicality for product assembly. Finally, in scenario analysis, a number of potential waste management interventions were composed, informed by current work streams in the facility, evidence from prior literature, and the results of the exploration of more frequent FLW causes. The results of different scenarios were analysed with respect to raw material and cost savings, to explore the potential opportunities for FLW reduction and increased circularity for the sector. Details of each scenario are shown in Table 6.

**Table 6:** Description of waste management interventions modelled in scenario analysis

Scenario	Description and assumptions
A	Base case: current manufacturing and waste management practices
B	Theoretically avoidable assembly processing losses are reduced by half, for example, through staff training, process and product design changes, or digital tracking^33,101^. It is assumed that ingredient utilisation remains the same, while processing losses reduce by 50% from the base case.
C	An alternative valorisation route for unused bread is found. Waste bread is diverted from animal feed towards reformulation or bioconversion, for example, to breadcrumbs or through fermentation^117,122^, and is thus no longer considered a waste stream. Assumes that all bread is separated in pure form suitable for valorisation.
D	Product visual specifications are eased so that fewer ingredients are rejected based on narrow visual tolerances. In this case, quality rejects at ingredient intake are still retained to ensure adequate product safety, while edible ingredients and finished products discarded in preparation or assembly stages are eliminated and all suitable components incorporated into the saleable product pool. It is assumed that secondary markets or food waste awareness campaigns are implemented to prevent increased consumer waste from product rejections^106,107^.
E	Combined case where interventions in B, C and D are implemented concomitantly; waste reductions across interventions are cumulative.

## Supplementary information

Supplementary Information

## Data Availability

The datasets generated and/or analysed during the current study are not publicly available due to commercial confidentiality, but are available from the corresponding author on reasonable request and with permission from Samworth Brothers Ltd.
