# Supplementary material for: Circling the sandwich: measuring food waste and its drivers in UK prepared sandwich manufacture
Source: NPJ Sci Food. 2026 Jun 27;10:316. doi: 10.1038/s41538-026-00948-8 (PMC13677716; doi:10.1038/s41538-026-00948-8)
Supplement: Supplementary file 1 [file 41538_2026_948_MOESM1_ESM.pdf]

Circling the sandwich: measuring food waste and its drivers in UK prepared sandwich manufacture – Supplementary Information

Table S1. Typical ranges of ingredient compositions for chicken salad sandwiches[1–5], into which the recipe examined in this work falls, and variation in ingredient usage recorded in the two sample production runs. Note: in the examined product, the mayonnaise component contains additional black pepper seasoning at 1 % of ingredient mass.

**Ingredient**	**Range of composition in similar retail sandwiches** Max.	Min.	**Variation recorded in sample manufacturing runs** Run 1 = 2576 units; Run 2 = 1944 units.			
**Chicken**	30%	25%		1.2%		
**Bread**	45%	41%		0.0%		
**Lettuce**	8%	4%		1.8%		
**Cucumber**	6%	5%		11%		
**Tomato**	8%	6%		4.3%		
**Mayonnaise**	13%	9%		8.0%		
**Total weight (g)**	**239**	**193**	**Total variation**	**1.8%**	**Total units in sample runs**	**4520**

References

1. Espinoza-Orias, N. & Azapagic, A. Understanding the impact on climate change of convenience food: Carbon footprint of sandwiches. *Sustainable Production and Consumption* **15**, 1–15 (2018).
2. ASDA. ASDA Chicken Salad on Malted Bread Product Page. https://www.asda.com/groceries (2025).
3. Morrisons. Morrisons Chicken Salad Sandwich Product Page. https://groceries.morrisons.com/ (2025).
4. Sainsburys. Sainsbury's Chicken Salad Sandwich Product Page. https://www.sainsburys.co.uk/gol-ui/groceries (2025).
5. Tesco. Tesco Chicken Salad Sandwich Product Page. https://www.tesco.com/ (2025).
